# Are we underestimating the impact of COVID-19 on children’s physical activity in Europe?—a study of 24 302 children

**DOI:** 10.1093/eurpub/ckac003

**Published:** 2022-01-12

**Authors:** Viktoria A Kovacs, Mirko Brandes, Thomas Suesse, Rok Blagus, Stephen Whiting, Kremlin Wickramasinghe, Anthony D Okely

**Affiliations:** Hungarian School Sport Federation, Budapest, Hungary; Leibniz Institute for Prevention Research and Epidemiology (BIPS), Bremen, Germany; School of Mathematics and Applied Statistics, University of Wollongong, Wollongong, Australia; Medical Faculty, Institute for Biostatistics and Medical Informatics, University of Ljubljana, Ljubljana, Slovenia; WHO European Office for the Prevention and Control of Noncommunicable Diseases, Moscow, Russia; WHO European Office for the Prevention and Control of Noncommunicable Diseases, Moscow, Russia; Early Start and Illawarra Health and Medical Research Institute, University of Wollongong, Wollongong, Australia

## Abstract

This repeat cross-sectional study investigated the impact of lockdown in Europe in Winter (January and February 2021) on children’s and adolescent’s physical activity (PA) and recreational screen time (RST), and compared PA to the lockdown in Spring 2020. An online survey was administered (*n* = 24 302; 6–18 years; 51.7% boys) in nine countries. PA and RST were assessed by 7-day recall. In total, 9.3% of children (95% confidence interval: 6.9–11.7) met WHO PA recommendation, which was half of the proportion observed in Spring 2020 [19.0% (18.2–19.9)]. Sixty percent exceeded the RST recommendations. This suggests that winter lockdown could have a more negative impact on PA than in spring.

## Introduction

Widespread concerns exist among public health professionals that children’s physical activity (PA) will decline due to the SARS-CoV-2 (COVID-19) pandemic and associated measures. Such declines in PA could pose greater concerns to children’s health in the long term than the risks of the COVID-19 infection. These concerns stem from data collected during the first lockdown period in March–May 2020.^1,2^ After battling severe outbreaks of COVID-19 infections, public health experts and policymakers need to respond to mitigate the long-term health effects. But does existing information provide good estimates on the decline in PA in children as a result of COVID-19?

Several studies, including our previous multi-national study of 8400 children,^3^ reported a low level of PA in Europe when the first safety measures were introduced in response to the global COVID-19 pandemic. Yet this could have potentially underestimated the real situation as spring 2020 was characterized by a particularly good period of weather, which is a well-known factor in promoting higher levels of PA among children.^4^ Thus, even if opportunities for PA were limited due to the COVID-19-related restrictions, children could go outside and be active. It can be hypothesized, however, that PA may have dropped more during the winter months of lockdown as children were less able to play outside and indoor opportunities were limited.

We conducted a follow-up survey in the winter period in nine European countries. The objective of this repeated cross-sectional study was to describe the PA and recreational screen time (RST) in a multi-national sample of European school children and adolescents during the period of winter lockdown and to compare the results for PA to our initial data from spring 2020. The *a priori* hypothesis was that winter lockdown would have more alarming impact on PA in European children and adolescents.

## Methods

This cross-sectional study was conducted among children and adolescents (aged 6–18 years) from nine European countries (Denmark, Germany, Hungary, Italy, Poland, Portugal, Russian Federation, Slovenia and Spain) between 26 January and 26 February 2021. The online survey was open in countries for 13–23 days. Data collection methods were standardized across countries. Target recruitment was a minimum of 200 children per country collected via convenience sampling. A 7-day recall measure was used to assess the PA and RST. Questions were taken from our previous survey,^3^ with several new items added to assess, among others, the perceived changes in PA between the first and second data collection periods. The final questionnaire contained 37 items. For older children (≥15 years), the questionnaire was self-administered. Parents of children aged <15 years were asked to help their child complete the questionnaire. PA (≥60 min/day) and RST (≤2 h/day) were considered sufficient according to the guidelines for children.^5^ Ethical approval was obtained by the leading partner and the study protocol was approved by the Scientific and Research Ethics Committee of the Medical Research Council in Hungary (Approval Number: IV/307-1/2021/EKU), as well as by each centre’s local ethics committee.

Survey data were obtained from the online platforms, pooled, cleaned and imported for analysis using R language for statistical computing (R version 4.0; R Core Team, Vienna, Austria). Descriptive data were presented as frequencies (%) with 95% confidence intervals (CIs, Wald-type) or means with corresponding 95% CI, respectively. Means and percentages have been adjusted for age by sex based on European Population counts from 2020 (except Russia using 2019 data), using post-stratification.^6^

## Results

Of the 28 273 respondents who completed the second survey, 3971 were excluded (missing consent: 1540; reported symptoms: 1472; reported chronic illness: 865 and missing PA data: 384) leaving a final analytic sample of 24 302 children [median age (interquartile range): 12 (9–14) years]. Due to the convenience sampling design, information on the response rate and non-responders’ characteristics were unavailable. Boys were slightly overrepresented (51%), as were participants living in urban areas (56.4%) and those from the Russian Federation (48.1%). In total, 2.7% of children reported to be in self-isolation or quarantine at the time of the survey (Supplementary table S1).

Country-based descriptive statistics for meeting the PA guideline and for RST are shown in table 1. Very few children and adolescents met the PA recommendation [9.3% [6.9–11.7)]. Less than two-third of participants [60.6% (55.8–65.4)] met the recommended 2 h/day of RST on weekdays, and less than half of participants [47.7% (41.8–53.5)] met the recommendation on weekend days.

**Table 1:** Key study variables by country in European children and adolescents (6–18 years; *n* = 24 302)

	Meeting 60-min MVPA guideline everyday^a^	Recreational ST— weekdays (hours)^b^	Meeting ≤2 h ST recommendation— weekdays^a^	Recreational ST— weekend (hours)^b^	Meeting ≤2 h ST recommendation— weekend^a^
Denmark (*n* = 1150)	11.02 (9.2–12.9)	3.5 (3.4–3.7)	33.8 (30.4–37.2)	4.3 (4.2–4.4)	21.1 (18.3–23.8)
Germany (*n* = 1203)	10.3 (7.1–13.6)	2.6 (2.5–2.7)	55.6 (51.7–59.5)	3.2 (2.9–3.4)	47.2 (43.4–50.9)
Hungary (*n* = 857)	12.2 (8.9–15.5)	2.2 (2.1–2.4)	65.6 (62.03–69.2)	3.04 (2.9–3.2)	43.8 (39.7–47.8)
Italy (*n* = 617)	4.2 (2.3–6.1)	2.3 (2.1–2.4)	63.03 (58.9–67.1)	2.5 (2.4–2.7)	55.9 (51.5–60.2)
Poland (*n* = 1990)	14.8 (12.9–16.5)	2.6 (2.5–2.6)	59.4 (57.2–61.6)	3.02 (2.9–3.1)	46.2 (43.9–48.5)
Portugal (*n* = 458)	4.0 (2.2–5.8)	2.8 (2.6–3.03)	53.9 (48.1–59.7)	3.6 (3.4–3.8)	35.7 (30.4–40.9)
Russian Federation (*n* = 11 686)	7.3 (6.3–8.3)	1.9 (1.8–1.9)	72.4 (70.7–73.9)	2.3 (2.2–2.3)	61.9 (60.1–63.6)
Slovenia (*n* = 5642)	12.6 (11.6–13.6)	2.2 (2.1–2.2)	66.5 (65.1–67.9)	2.4 (2.3–2.4)	60.2 (58.8–61.7)
Spain (*n* = 699)	7.3 (5.1–9.4)	1.7 (1.6–1.8)	75.2 (72.2–78.1)	2.4 (2.3–2.6)	57.4 (53.9–60.9)
Combined (*n* = 24 302)	9.3 (6.9–11.7)	2.4 (2.2–2.6)	60.6 (55.8–65.4)	2.9 (2.7–3.2)	47.7 (41.8–53.5)

a % (95% CI for %).

b Means (95% CI for means). MVPA, moderate-to-vigorous physical activity; ST, screen time. Means and percentages have been adjusted for age by sex based on European Population counts from 2020 (except Russia using 2019 data), using post-stratification. Combined refers to combined percentages or means, based on a random effect meta-analysis using the R package meta.

Around one-quarter of the respondents [25.0% (19.3–30.7)] reported being less active now and a similar proportion [23.9% (18.0–29.8)] reported spending less time in vigorous-intensity PA compared with the first wave of the pandemic in Spring 2020.

## Discussion

The purpose of this study was to assess the impact of the COVID-19 pandemic on PA and RST in European school-aged children in winter and to compare the results for PA to our previous data collected during the initial period of COVID-19 restrictions in 2020. Our data revealed a low amount of PA and high levels of RST during winter lockdown among European school-aged children. Compared with what we reported previously and from other studies conducted during the same period,^1–3^ the impact of winter lockdown on children’s activity was more striking in this large sample of European school children. The percentage of children who met the WHO PA recommendation decreased by half compared with what was observed in Spring 2020.^3^ This large drop cannot be explained solely by the differences in the characteristics of the respondents as in the second sample children were younger and fewer participants reported being in self-isolation than during the initial survey period (Supplementary table S1). A limitation of our study is that we could not compare our findings for screen time because in spring 2020 we asked respondents about their *total* screen time (educational and recreational), whereas in winter 2021 we asked participants separately about their *educational* and RST.

Policy makers and stakeholders need to be aware and understand this to be able to initiate proper mitigation measures. Of public health concern is that these short-term changes in behaviour in reaction to COVID-19 may become permanent. Although available data from repeated surveys are limited,^7^ signs are concerning. Data from one other follow-up study showed that fewer children met the PA recommendations in October 2020 than in April 2020; however, the difference was not as remarkable as in our study.^7^

Because this is an unprecedented situation, it is unknown how to best respond. But efforts must be made to advocate for an active lifestyle. It has been shown among adults that those who are adequately active were less likely to be infected with COVID-19 or to suffer significant co-morbidities.^8^ As new strains such as the Delta variant become widespread and affect more younger people, it is important that, in addition to promoting vaccination, healthy levels of PA are also encouraged. Family, school and community support are important to ensure that this dramatically low level of PA does not become the new normal. Stakeholders are required to take responsibility for promoting healthy levels of PA and RST during children’s academic and leisure time. A focus on the promotion of healthy levels of these behaviours must be included in efforts to respond to the pandemic.

## Supplementary Material

ckac003_Supplementary_Data
