# Supplementary material for: Are we underestimating the impact of COVID-19 on children’s physical activity in Europe?—a study of 24 302 children
Source: Eur J Public Health. 2022 Jan 12;32(3):494–6. doi: 10.1093/eurpub/ckac003 (PMC9159340; doi:10.1093/eurpub/ckac003)
Supplement: ckac003_Supplementary_Data [file ckac003_supplementary_data.docx]

**Supplementary Table 1.** Characteristics of the two analytic samples and key study variables.

|  | 1st round (May-June, 2020) | 2nd round (Jan-Feb, 2021) |
| --- | --- | --- |
| Sample size (n) | 8,395 | 24,302 |
| Median age [IQR] (years) | 13 [10-15] | 12 [9-14] |
| % of boys | 47 | 51.7 |
| % of urban residents | 57.6 | 56.4 |
| % in self isolation | 15.5 | 2.7 |
| % of meeting 60-min MVPA guideline everyday (95% CI for %) | 19.0% (18.2 to 19.9) | 9.3% (6.9 to 11.7) |
| % of meeting ≤2h ST recommendation on weekdays (95% CI for %)* | 30.5% (29.5 to 31.6) | 60.6% (55.8 to 65.4) |
| % of meeting ≤2h ST recommendation on weekdays (95% CI for %)* | 36.3% (35.25-37.3) | 47.7% (41.8 to 53.5) |

MVPA=moderate-to-vigorous physical activity; ST=screen time. *Note: In the 1st round ST refers to TOTAL screen time, while in the 2nd round ST refers to RECREATIONAL screen time.
